# On the variability and dependence of human leg stiffness across strides during running and some consequences for the analysis of locomotion data

**DOI:** 10.1098/rsos.230597

**Published:** 2023-08-23

**Authors:** Alessandro Maria Selvitella, Kathleen Lois Foster

**Affiliations:** ^1^ Department of Mathematical Sciences, Purdue University Fort Wayne, 2101 East Coliseum Boulevard, Fort Wayne, IN 46805, USA; ^2^ eScience Institute, University of Washington, 3910 15th Avenue Northeast, Seattle, WA 98195, USA; ^3^ NSF-Simons Center for Quantitative Biology, Northwestern University, 2200 Campus Drive Evanston, IL 60208, USA; ^4^ Department of Biology, Ball State University, 2000 West University Avenue, Muncie, IN 47306, USA

**Keywords:** biomechanics, kinematic and kinetic data, multi-stride dependence structure, variability, leg stiffness, running

## Abstract

Typically, animal locomotion studies involve consecutive strides, which are frequently assumed to be independent with parameters that do not vary across strides. This assumption is often not tested. However, failing in particular to account for dependence across strides may cause an incorrect estimate of the uncertainty of the measurements and thereby lead to either missing (overestimating variance) or over-evaluating (underestimating variance) biological signals. In turn, this impacts replicability of the results because variability is accounted for differently across experiments. In this paper, we analyse the changes of a couple of measures of human leg stiffness across strides during running experiments, using a publicly available dataset. A major finding of this analysis is that the time series of these measurements of stiffness show autocorrelation even at large lags and so there is dependence between individual strides, even when separated by many intervening strides. Our results question the practice in biomechanics research of using each stride as an independent observation or of sub-selecting strides at small lags. Following the outcome of our analysis, we strongly recommend caution in doing so without first confirming the independence of the measurements across strides and without confirming that sub-selection does not produce spurious results.

## 1. Introduction

In biomechanical studies of human or animal locomotion, in particular in the analysis of terrestrial gaits, it is common practice for researchers to take measurements and compute variables of interest for each stride and then average or analyse the values of those variables across strides, often considering each stride an independent and identically distributed (iid) observation [1–5]. As a general rule, an effort is made to keep all possible strides available. Most of the experiments in the comparative and human locomotion literature are based on sample sizes of less than 50, and often even smaller, less than 10 (e.g. [6–10]), and so researchers want to keep as much information as possible from the experiments performed. Enlarging the study is often either costly or not feasible because of characteristics of the species (e.g. rapid growth rate, challenging/rare behaviours) or the conditions of the equipment or because the population to be sampled does not allow for larger testing procedures. For example, enlarging a sample for a comparative study could mean going back to the field and taking a new sample, a very cumbersome path to follow [11]. Extending the data collection for longer periods of time would not be a solution as that would require complicating the model and, therefore, the extra inflow of data may not keep up with the extra-complexity required to model a growth study. These small sample issues for biomechanical studies are an ubiquitous feature of the field, even in this big data era. Note that the practice of treating the strides as independent is diffuse across different areas of the locomotion literature. Researchers studying humans [3,6,12–14], bipedal birds [5,8,10,15,16], lizards [9,17–20] and other animals as well [2,4,21–23], estimate parameters of biomechanical relevance by averaging measurements across consecutive locomotor cycles and using statistical methods based on the independence hypothesis.

Sometimes, to avoid the extra challenges posed by correlated data, researchers choose to only use measurements from every two or three strides for their average estimate [12,24]. In extreme cases, only one single stride per trial is considered [25–27]. This strategy has distinct disadvantages: it reduces the total number of strides that can be used in the study and disrupts the natural correlation structure present in the data, between strides. In many cases, researchers do not rely on the structure of the data to decide how many strides to drop or how many can be considered independent and, for those which are not independent, to determine which type of dependence structure there is [12,24–26]. When steady-state locomotion is being studied, the first and last strides from a given trial are often excluded or take place off-camera and so are not recorded [1]. Such decisions to exclude strides are, in general, made by the single researcher and they are not standardized across laboratories. Using personalized stride exclusion criteria does not only risk to preclude the correct reporting of the results of the experiments, to miss the opportunity to learn about the irregular biomechanics at the beginning of a cyclical motion, and to jeopardize the replicability of the experiments across laboratories, but also is questionable from the data science perspective. It is hard to justify the decision to exclude some strides from analysis simply because they might be dependent, particularly considering that any dependence present in a time series may arguably be the most important feature.

The main objectives of this manuscript are, first, to underline that it is incorrect to not account for variability of measurements across strides in the analysis of data from locomotion experiments and, then, discuss the possible consequences of doing so. For these objectives, we concentrate on one relevant example and study it extensively, but we believe that our considerations are valid in general for terrestrial locomotion experiments which involve multi-stride data. We study the variability of human leg stiffness across strides during running on a treadmill at different velocities and use a publicly available dataset [28] to illustrate our argument. Note that this topic has not received extensive attention in human locomotion studies and so our results also provide new insights on this important problem.

Most commonly, human running is modelled as a *spring-mass*/*spring loaded inverted pendulum* (SLIP) [29–32]. This model is reductionist and tries to extract the most fundamental features of the complex dynamics of human running with a low-dimensional system of ordinary differential equations describing bouncing gaits. The resulting model has qualitative validity in the sense that trajectories of the SLIP system resemble those of bipedal runners and many other multi-pedal runners ([29,30,33] and the large body of literature that cites these papers). As far as we know, there are no proven mathematical theorems that guarantee under which regimes (e.g. small joint angles, low horizontal velocity) the high-dimensional dynamical system describing an animal running can be accurately determined by a low-dimensional model, such as the two-dimensional SLIP system. One of the consequences of the lack of guarantees of these reductionist models is the difficulty in establishing the best methodology for estimating parameters of interest, such as leg stiffness, through models like the SLIP model [34–38]. The basic idea of this *spring-mass* model is to use a linear spring that follows Hooke’s law and compute the stiffness by dividing the maximum (max) vertical ground reaction force (GRF), typically measured using kinetic data from force platforms, and the compression of the leg deduced using kinematic data, such as the position of the centre of mass (CoM) [29,30].

Considerable discussion in the literature has centred around how to calculate leg stiffness during human running and the validity of the various methods has been questioned [34]. Some methods include the measurements of the leg length and GRFs at touch down, such as those employed in [35,36,39], and the use of a mechanical arm to track the movement of the CoM [40]. In some situations, the stiffness is a parameter estimated from a chosen model and, in such cases, it simply comes from the model assumptions rather than existing as a biological parameter itself. In the SLIP model, the stiffness represents some sort of summary measure of the properties of the individual which appears to globally possess a *spring-mass* behaviour, but it is not a direct measurement of the elasticity of any specific muscle/tendon. In [41], the authors noted that the stiffness of the leg cannot be calculated directly neither in humans nor in bipedal birds and that an effective leg stiffness can be estimated only if assuming that the entire body behaves like a spring-mass system during running. It does make sense to estimate such a parameter from the data, but the interpretation of such an estimate needs to be used with caution. As mentioned, there are many methods used to estimate stiffness, some of them described in [42] (see also [43]), in which authors compare five methods for the estimation of the stiffness parameter based on kinematic and kinetic data. The authors in [34] noticed that reductionist models often require untested assumptions for their mathematical analysis to be carried out, but that those assumptions are not tested and comparisons with the consequences derived from different assumptions are rarely made. Furthermore, [44] noted that the method in [30] underestimated leg stiffness compared to their own method and that, in [30], the authors did not note a dependence of leg stiffness with velocity, whereas [42] reported similar leg stiffness values to those following the method proposed in [30]. Also, the authors in [34] compared leg stiffness values measured using direct kinematic and kinetic methods with leg stiffness values calculated using the most common methods present in the literature. They found that stiffness estimates are highly variable across methods and studies. In addition to the challenge of estimating parameters of interest, a further difficulty comes from the fact that, in most models, the human body is assumed to be rigid, but GRFs do not directly act on the CoM of the body. Such a simplification neglects dissipative forces in the transmission of the GRFs from the centre of pressure of the foot in contact with the ground to the CoM. There is actually not a strong argument to assume that the GRFs act on the CoM directly [45].

The authors in [46] found that human runners adjust their leg stiffness [36,47] to accommodate changes in surface stiffness, allowing them to run with similar dynamics on different surfaces. Several studies have investigated the way in which leg stiffness is adjusted to accommodate changes in ground level. In particular, the authors in [48] studied how runners modulate ankle and knee joint stiffness, finding, among their other results, that the ankle joint stiffness depends on the vertical height of a step, similar to the global leg stiffness. In [49], the author addressed the problem of joint level compliance during human walking and how joint stiffness is modulated during human walking on flat surfaces, inclined surfaces, and stairs. The results found that stiffness estimation was much lower than those found in running ([50]; see also [51,52]. As far as we know, there has not been an extensive study of how stiffness changes across strides at different running velocities. Leg stiffness is also of interest in animal biomechanics [34,53–56] (and papers referring to these) and the variability of leg stiffness as a function of speed has been a source of discussion in comparative biomechanics as well. Some experimental studies have reported that animal leg stiffness is independent of speed [36,46,57].

It is a very hard problem to determine how age influences the changes across strides of leg stiffness while running at different velocities [58]. Even when considering a single individual, there may be many confounding factors (e.g. history of injury, typical footwear etc.) which need to be taken into consideration to properly address the problem. Note that being able to understand how the dynamics of older versus younger individuals changes, in particular how leg stiffness is modulated through stride as a function of age, can have important consequences in fields such as kinesiology, rehabilitation and prosthetics. With longer life expectancy, many more people encounter orthopaedic problems [59], including those with moving, particularly those related to optimal running modes. How these problems arise is still poorly understood. Muscle stiffness has been reported to be associated with the reduction of force generation capability of quadriceps in older populations [60]. Such a decrease in muscle strength has been associated with the slowing of movements in elderly people, together with this rigidity or increased stiffness of muscles ([60]; see also the work in [58,61]. None of these results seem to address if there is a significant change of leg stiffness through strides or prolonged running experiments across age groups. The problem of how changes in stiffness across strides depend on mass is complex as well, as there are no simple physical principles that help in the modelling of such a change. Many experiments and analyses need to be done. Some studies have investigated how leg stiffness scales with respect to mass in bipedal and quadripedal mammals in both running and hopping, using experimental and simulated data [35,62–65], but, as far as we know, none have discussed how mass impacts the change in stiffness across strides.

In summary, the goals of the paper are the following: (i) to determine the influence that neglecting the dependence structure of strides can have on the results from the analysis of kinetic and kinematic data, using measurements of leg stiffness averaged across strides as a running example; and (ii) to provide a better understanding of the variability across strides of human leg stiffness during treadmill running.

## 2. Methods

In this section, we describe the methods we used for our analysis.

### 2.1. Dataset

The dataset that we used is publicly available [28]. As explained in the data repository [28], the dataset collects multivariate time series of the trajectories of signals from sensors applied to the human body while running on a treadmill at 2.5 m s^−1^, 3.5 m s^−1^ and 4.5 m s^−1^. The time series include both kinematics and kinetics of 28 subjects and also includes metadata with age and mass among the variables considered. Although, we will analyse only the dynamics in the sagittal plane, the data comprise three-dimensional coordinates collected from a motion-capture system.

### 2.2. Pre-processing of the data

Before starting the analysis, we pre-processed the data. The frequency of collection of kinematic and kinetic measurements were different; the time series of the forces were collected at 300 Hz while the time series of the markers were collected at 150 Hz. For our analysis, we sub-selected every other datapoint in the ground reaction force data and paired them with the full kinematic data. During the aerial phase, the vertical components of the forces exerted on the foot is zero, and so the only force applied to the CoM is the gravitational force. This is true because the foot is not touching the treadmill and so force platforms cannot record any force. We excluded the aerial phase from our analysis, as we are interested in understanding the elastic properties of the leg and in the aerial phase those elastic forces are zero. To do so, we extracted from the full kinematic and kinetic time series of each subject the measurements of the forces for all instants constituting only the stance phases as determined by the condition that the vertical force recorded was positive (*F*_*y*_ > 0). Furthermore, the kinematic data consisted of several time series of markers placed on the body of the participants recorded during the experiments. Not all of these markers were useful for understanding the reaction of the individual (e.g. change in leg stiffness) to the collision of the foot onto the treadmill. As we only needed the information about the CoM, which is located approximately at the height of the hips, we retained and averaged only the coordinates of the left and right markers (in the sagittal plane with positive *x* concordant with the running direction and positive *y* in the direction foot-to-head) that were closest to this position: the *anterior superior Iliac spine*, the *posterior Iliac spine* and the *Iliac crest* coordinates.

Then, we extracted each single stride and stored the time series of these strides in separate variables. Finally, the coordinate system in which the dataset was collected was centred at an origin static with respect to the treadmill. Therefore, we performed a change of variables that allowed us to recentre the data: *x*′ = *x* + v*t, *y*′ = *y* + v*t with (*x*, *y*) the coordinates with respect to the treadmill origin, *t* the time index and *v* the velocity of the treadmill in that trial. Note that the way in which we computed the CoM is just one of the ways available in the literature. For example, the authors in [66] estimated the vertical and relative adaptation of the CoM using three different markers: the coordinates of the fifth lumbar vertebrae and the seventh cervical vertebrae and the average of the coordinates of the right and left greater trochanter from the upper leg.

### 2.3. Calculations of leg stiffness

Leg stiffness can generally be defined as *k* = ∂*F*/∂*L*, with *F* being the vertical component of the GRF and *L* the leg length during the stance phase [42]. As mentioned in [42,43], there are many ways to define that quantity, both in a linear and nonlinear way. Here, we do not argue in favour or against any of the methods, but we consider a sub-selection of those methods. We compute the leg stiffness in two different ways:

— *method 1*: the maximal leg stiffness *k*_max_ was computed as the resultant force in the direction of the leg spring *F*_leg_ divided by the leg compression Δ*L* : *k*_leg_ = *F*_leg_/Δ*L* (see for example, [34]). *F*_leg_ was computed as the value of the vertical GRF, *F*_*y*_ , computed at the lowest height *y* of the centre of mass, after correcting *F*_*y*_ for the weight of the individual. The leg compression Δ*L* was computed as:
  $$\Delta L=\max(y)-\min(y)+l_{0}*\left(1-\sqrt{1-{\left(\frac{\max(x)-\min(x)}{\left(2*l_{0}\right)}\right)}^{2}}\right).$$
  Here, max (*y*), min (*y*) are the maximal and minimal vertical position, with respect to the height of the centre of mass during the stance phase, while max (*x*), min (*x*) are the maximal and minimal *x* coordinates of the centre of mass during that particular stance phase and *l*_0_ is the height of the CoM at rest. Here, *x* and *y* are calculated after the change of variable performed in the pre-processing step; and
— *method 2*: we computed *k*_OLS_ using the ordinary least-squares method for a linear model without intercept. The independent variable was the GRF defined using a *spring-mass* model:
  $${\mathrm{GRF}}_{y}=y*\left(\frac{l_{0}}{\sqrt{x^{2}+y^{2}}-1}\right),$$
  and the dependent variable was the vertical component of the recorded GRF plus the weight (m*g, with *g* = 9.81 m s^−2^) of the individual.

### 2.4. Hypothesis tests for stride independence

In this subsection, we describe the procedures that we followed to test the hypothesis *H*_0_: *strides are independent* versus *H*_*a*_: *strides are not independent*, and in particular the hypothesis that the leg stiffness measurements *k*_OLS_, *k*_max_ are or not stride independent. Note that if summary statistics calculated from different populations are dependent, then the two populations are dependent as well [67]. Therefore, the dependence of the stiffness on stride indicates a dependence of the stance phases themselves within experimental trials. We concentrated on tests which measure the possibility of autocorrelation in the time series of the stiffness measurements. All tests were performed at significance level *α* = 0.05.

Yule test

The simplest method to test for autocorrelation in a time-series dates back to Yule [68,69]. For a given sample $Y_{1},\ldots \;,Y_{n}$, the population autocorrelation function (ACF) *ρ*_*Y*_(*k*) is estimated by the sample ACF ${\hat{\rho }}_{Y}(k)$ for k=1,2,…. Note that the ACF ${\hat{\rho }}_{Y}(k)$is well defined for all *t*, *k* = 1, 2, …, even for non-stationary processes (but with finite first and second moments). The ACF is given by

$${\hat{\rho }}_{Y}(k)\;:=\frac{{\hat{\gamma }}_{Y}(k)}{{\hat{\gamma }}_{Y}(0)},\;{\hat{\gamma }}_{Y}(k)=\frac{1}{n}\sum_{t=1}^{n-k}(X_{t}-{\bar{X}}_{T})(X_{t+k}-{\bar{X}}_{n}),\;t=0,1,\ldots ,n-1.$$

The asymptotic distribution of the sample autocorrelations ${\hat{\rho }}_{Y}(k)$ for various lags *k* = 1, 2, … for a general stationary linear process [70,71] takes the form

$$\sqrt{n}({\hat{\rho }}_{Y}(1)-\rho_{Y}(1),\ldots ,{\hat{\rho }}_{Y}(m)-\rho_{Y}(1))\to N(0,W),$$

as *n* → +∞ with *W* given by Bartlett’s formula [72], which, in the case of a null hypothesis given by iid random variables, reduces to the identity matrix *W* = *Id*_*m*_ and so

$$\sqrt{n}({\hat{\rho }}_{Y}(1),\ldots ,{\hat{\rho }}_{Y}(m))\to N(0,Id_{m}),$$

with *Id*_*m*_ the identity matrix in dimension *m* and *m*, the maximum lag considered. Therefore, to test the hypothesis

$$H_{0}\;:\;\rho_{k}=0\;vs\;H_{A}\;:\;\;\mathrm{otherwise},$$

at level *α*, it is enough to verify that $|{\hat{\rho }}_{Y}(k)|>z_{\alpha /2}/\sqrt{n}$ with *n* the size of the sample observed [73].

There is a vast literature on testing for significant autocorrelation in time series, starting from tests not only at one single lag *k*, but for a series of lags, such as $H_{0}\;:\;\rho_{Y}(1)=\cdots =\rho_{Y}(m)=0$ [74,75]. We considered these cases too.

Ljung-Box test for independence

The Yule test detects if there are correlations at single lags. Sometimes single autocorrelations oscillate and dependency in a time series can be detected only by grouping autocorrelations together and verifying their magnitudes. The Ljung-Box test [75] aims at detecting if a group of autocorrelations of a time series is significantly different from zero. The Ljung-Box test statistic is given by:

$$Q=n(n+2)\sum_{k=1}^{m}\frac{{\hat{\rho }}_{Y}{\left(k\right)}^{2}}{n-k},$$

where *n* is the sample size and *m* is the number of lags being tested. We performed a Ljung-Box test with maximum lag 40 at each velocity and for both measures of stiffness, *k*_max_ and *k*_OLS_. We collected the *p*-values for each of the tests. We considered a Bonferroni-type adjustment with significance level *α*/*N* with *α* = 0.05 and *N* = 28. As a follow-up to the Ljung-Box test, we used linear models to determine how the dependency of stride was influenced by age and mass, using the *p*-values of the Ljung-Box test for maximum lag equal to 40 as the dependent variable and age and mass as independent variables.

Linear mixed effects models

We analysed how the stiffness was influenced by stride and velocity (see appendix A 4). To do so, we developed several linear mixed effects models (LMEMs) with subject dependency as a random effect [76]. We developed LMEMs including the velocity as a fixed effect, but also separate models for each single velocity. We restricted our attention to the case where *β*_1,*j*_ = *β*_1_, namely the case in which only the intercept of the model includes random effects accounting for between subject variations. More specifically, we considered the following models for both *k* = *k*_max_ and *k* = *k*_OLS_:

$$k_{ij}=\beta_{i,0}+\beta_{1}S_{ij}+\varepsilon_{ij},$$

for each of the three velocities (*v* = 2.5 m s^−1^, *v* = 3.5 m s^−1^ and *v* = 4.5 m s^−1^). Here, *j* = 1, …, 28 and *i* = 1, …, *n*_*j*_ with *n*_*j*_ the number of strides for subject *j* for each single velocity. *S*_*ij*_ represents stride number *j* for subject *i*. Furthermore, we considered the models with velocity included for both *k* = *k*_max_ and *k* = *k*_OLS_:

$$k_{ij}=\beta_{i,0}+\beta_{1}S_{ij}+\beta_{2}v_{ij}+\varepsilon_{ij},$$

for each of the three velocities. As before, *j* = 1, …, 28 and *i* = 1, …, *n*_*j*_ with *n*_*j*_ the number of strides for subject *j* across velocities. Larger models including random effects affecting the inclination were not considered because, in such models, the estimation procedure was unstable, given that the complexity of those models was too high with respect to the sample size available in this study. Parameter estimation was performed using the restricted maximum likelihood criterion [77,78]. We fitted all LMEMs using the R-package *lmer* (see [79]).

## 3. Results

In this section, we outline the results of our analysis.

### 3.1. Dependence of stiffness across strides within individual subjects

**Figure 1.**
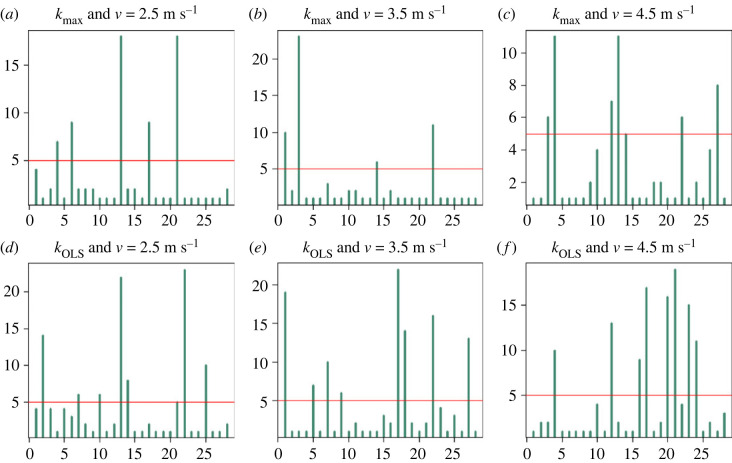
Yule test showing autocorrelation across strides at *v* = 2.5 m s^−1^ (*a*,*d*), *v* = 3.5 m s^−1^ (*b*,*e*), and *v* = 4.5 m s^−1^ (*c*,*f*) for *k*_max_ (*a*–*c*) and *k*_OLS_ (*d*–*f*). The *y*-axis of each plot corresponds to the lags, while the *x*-axis indexes the subjects. The vertical bars represent the largest lag which shows correlation across strides for each of the 28 subjects, while the horizontal line is fixed at a lag of 5 for illustrative purposes. In each of the conditions and for both *k*_max_ and *k*_OLS_, there is correlation across strides for a relevant number of subjects.

**Figure 2.**
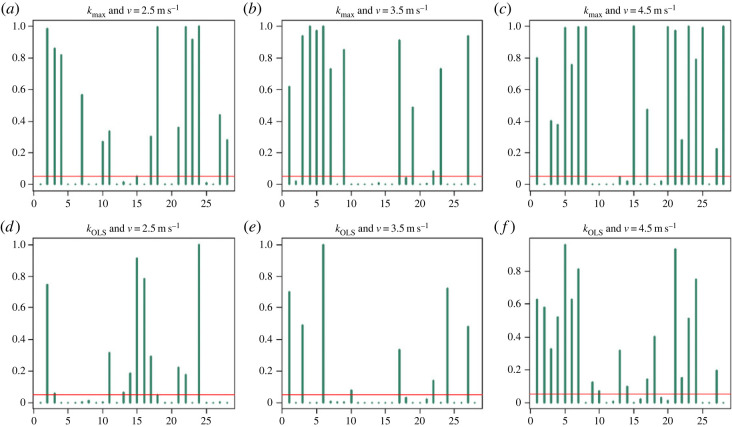
Ljung-Box test showing dependence across strides for the three velocities, *v* = 2.5 m s^−1^ (*a*,*d*), *v* = 3.5 m s^−1^ (*b*,*e*), and *v* = 4.5 m s^−1^ (*c*,*f*), and the two measures of stiffness, *k*_max_ (*a*–*c*) and *k*_OLS_ (*d*–*f*). The *y*-axis of each plot corresponds to the Ljung-Box *p*-value, while the *x*-axis indexes the subjects. The vertical bars represent the *p*-value of the Ljung-Box test for maximum lag equal to 40 for that subject, while the horizontal line is fixed at the level of significance (*α* = 0.05) for illustrative purposes.

In all of the six conditions analysed (two stiffness measures and three velocities), the Yule test revealed the presence of significant autocorrelation in the stiffness measurements that persists across strides even for lags larger than 5 in 5/28, 4/28, 6/28, 7/28, 8/28 and 8/28 subjects (figure 1, panels *a*–*f*, respectively). With the notation ‘*X/Y*’, we mean ‘*X* out of *Y*’. Note that a large number of locomotion studies consider each stride as independent and so, even just correlation at lag 1 would be a significant finding. The Ljung-Box test also found significant dependence across strides for many individuals. It determined that 11/28, 12/28, 8/28, 13/28, 16/28 and 6/28 individuals (figure 2, panels *a*–*f*, respectively) showed a dependence in stiffness across strides (only for those up to 40 lags apart) and so the strides are not iid; in particular they are not independent.

### 3.2. Dependence of strides with age and mass

**Figure 3.**
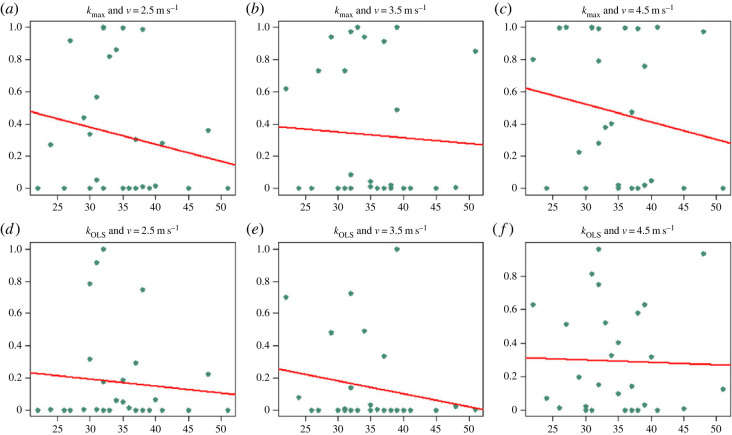
Dependence of the Ljung-Box test *p*-value with age. The *y*-axis represents the *p*-value of the Ljung-Box test at max lag 40, while the *x*-axis represents the age of the corresponding individuals. All of the plots show a decreasing trend but none of the relationships were statistically significant (p=0.3574,0.7737,0.3982,0.6224,0.3203 and 0.8704 in (*a*–*f*), respectively).

**Figure 4.**
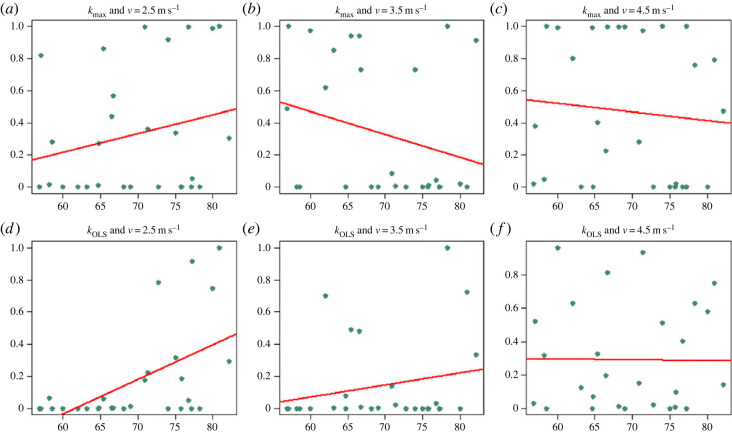
Dependence of the Ljung-Box test *p*-value with mass. The vertical axis represents the *p*-value of the Ljung-Box test at max lag 40, while the *x*-axis represents the mass of the corresponding individuals. Only one of the relationships was statistically significant (p=0.2372,0.1779,0.6282,0.002395,0.279 and 0.9537 in (*a*–*f*), respectively).

The analysis of age dependence of the Ljung-Box test indicates an increased dependence across strides with increasing age, but the estimates show high variability and none of the tests rejected the null hypothesis of *p*-value independent of age (p=0.3574,0.7737,0.3982,0.6224,0.3203 and 0.8704 in figure 3*a*–*f*, respectively. In the analysis of mass dependence of the Ljung-Box test *p*-values, only one test rejected the hypothesis of independence (figure 4*d*: *k*_OLS_, *v* = 2.5 m s^−1^) (p=0.2372,0.1779,0.6282, 0.002395,0.279 and 0.9537 in figure 4*a*–*c,e,f*, respectively).

### 3.3. Dependence of stiffness with stride and speed

The LMEMs for *k*_max_ and *k*_OLS_ with stride and velocity as fixed effects showed significant dependence of stiffness with both stride and velocity (tables 1 and 2). When we considered separate models per velocity, all models for *k*_max_ showed significant dependence across stride, whereas for the models for *k*_OLS_, only the model for *v* = 4.5 m s^−1^ showed significant dependence (see tables in appendix A 4).

**Table 1.** Results from linear mixed effects models with *k*_OLS_ as the measure of stiffness. (Stride and velocity were fixed effects, and subject was a random effect. Bold indicates *p* < 0.05.)

*k*_max_: linear mixed effects model					
fixed effects	estimate	s.e.	d.f.	*t*-value	*p*-value
(intercept)	15671.077	330.367	33.393	47.435	**<0.0001**
stride	−6.628	0.923	7171.831	−7.181	**<0.0001**
speed	279.633	28.404	7171.027	9.845	**<0.0001**

**Table 2.** Results from linear mixed effects models with *k*_OLS_ as the measure of stiffness. (Stride and velocity were fixed effects and subject was a random effect. Bold indicates *p* < 0.05.)

*k*_OLS_: linear mixed effects model					
fixed effects	estimate	s.e.	d.f.	*t*-value	*p*-value
(intercept)	19542.831	560.299	30.892	34.879	**<0.0001**
stride	−2.850	1.258	7171.518	−2.266	**0.0235**
speed	1261.722	38.711	7171.017	32.593	**<0.0001**

## 4. Discussion

Our analyses demonstrate that individual strides, even when separated by several intervening strides, cannot be assumed to be independent, unless proven otherwise. Therefore, our analysis leads to the recommendation that researchers test for dependence of strides in locomotion studies with multiple strides or consider consecutive strides as dependent. Below, we discuss several aspects of this result.

### 4.1. Non-independence of strides

The Yule tests that we performed underlined that correlation across strides is present even at large lags (figure 1). We detected the presence of correlation of the stiffness across strides at lags over 10 for at least one of the subjects in all the scenarios that we analysed (*k*_max_, *k*_OLS_ for *v* = 2.5 m s^−1^, 3.5 m s^−1^, 4.5 m s^−1^). Given a single subject and some measurements of biomechanical parameters (e.g. CoM height, mass, age), it is not simple to determine *a priori* if the measurements across strides will be independent or not, so rigorous statistical hypothesis testing is needed. Dependence of locomotion behaviour across strides seems to be a diffuse phenomenon in our sample population as correlation was detected for many individuals in multiple conditions. This dependence structure of strides during the running gait was confirmed by a lag-aggregate analysis performed with the Ljung-Box test with a maximum lag of 40 (figure 2). In some of the conditions analysed, close to 50% of the individuals performed running trials with strides that were not independent. This dependence seems greater at higher velocities, which could potentially be explained by the central nervous system having insufficient time at higher velocities to respond (i.e. alter stride characteristics) fast enough to sensory feedback from environmental perturbations. In such situations, the central nervous system would rely more heavily on preflexes and on pre-determined strategies for optimal locomotion, decentralized modes, and feedforward instead of feedback control [80]. Such a scenario would result in broad similarities in the characteristics of long time series of consecutive strides. In fact, relying on centralized feedback circuits would seem to be disadvantageous because of the intrinsic time delays, especially for locomotion at high speed [80]. This explanation seems in agreement with the very fundamental structure of models such as SLIP, which show passive stabilization properties based on preflexes [80].

### 4.2. Stride-dependence across ages, masses, and velocities

All six conditions analysed hint at a decreasing trend of the *p*-value of the Ljung-Box test with age, but possess quite a high variability across individuals, especially in relative terms for those individuals with ages between 30 and 40 years, and so no *p*-values were found significant at level *α* = 0.05 (see figure 3). This finding is in agreement with the results of [34]. Note that it is possibly harder to observe a significant relationship between age and stiffness over the limited age range represented in this dataset. Speculatively, having a range of ages spanning life periods from childhood to older ages would help detect a stronger relationship and determine what role the interaction between reaction time and age play in the locomotor behaviour across strides. Note that we are still quite far from having an accurate neuromechanical model of human locomotion and, as far as we know, there are few results on full neuromechanical models of animal locomotion available, most of which concentrate on insects [80,81]. Speculatively, an elderly individual with respect to a younger individual running at the same speed might pay more attention at each single step and so she/he might decouple the motion of one stride from the next, which in turn would result in a lack of autocorrelation in the time series of leg stiffness or other variables calculated per stride. Given the limits of the data available (e.g. small sample size per age-range), we did not try to optimize the model and merely chose the simplest possible hypothesis class (univariate linear regression; figure 3).

Mass was not correlated with the *p*-value of the Ljung-Box test for any condition except one, the case of *k*_OLS_ and *v* = 3.5 m s^−1^ (figure 4). We do not believe the significance of this one single test is particularly meaningful and we recommend also in this case further investigation with improved sampling across a wider range of masses to better determine the relationship between mass and stride dependence during running. It is largely unknown how to describe the complex relationship between age, mass, and other variables that can come together to contribute to determining stiffness. Altogether, our analysis of the relationship between variables such as age and mass with the modulation of leg stiffness across strides while running is inconclusive. Still, it can be considered at least exploratory and makes a case for the need for further investigation into this problem.

The LMEMs determined a strong effect of both stride and velocity on leg stiffness. The models with both stride and speed as fixed effects showed statistically significant correlation at level *α* = 0.05 (see tables 1 and 2). The models for each fixed velocity were all significant other than the model with *k*_OLS_ and *v* = 2.5 m s^−1^ (see appendix A 4 for more details about this). The dependence on velocity that we observed seem in agreement with the findings of [44], who found that the running velocity influences leg spring stiffness. However, the authors in [44] did not analyse the effect of consecutive strides on stiffness. This part of our analysis solidifies the understanding that measurements of stiffness across strides (and so strides themselves) are not independent.

### 4.3. Impact of ignoring dependence across strides

The variance of a sample mean from *Y*_1_, …, *Y*_*n*_ observations, which are iid, from a population with *E*[*Y*_1_] = *μ* and Var(*Y*_1_) = *σ*^2^ is given by $\mathrm{Var}[\bar{Y}]=\sigma_{Y}^{2}/n$. This formula is no longer valid when there are correlations between observations. For example, in the case in which a time series is observed from a stationary stochastic process, we have:

$$\mathrm{Var}[\bar{Y}]=\frac{\sigma_{Y}^{2}}{n}\left[1+2\sum_{k=1}^{n}\left(1-\frac{k}{n}\right)\rho_{k}\right],$$

with *ρ*_*k*_ the autocorrelation function at lags k=1,…,n. If we take the ratio between these two variances (the one for a general stationary process and the iid one), we get a quantity which contains information about the gain/loss of variance caused by neglecting possible autocorrelations in the data. This variance-to-variance ratio *R*(*ρ*_1_, …, *ρ*_*k*_) is given by

$$R(\rho_{1},\ldots ,\rho_{k})\;:=1+2\sum_{k=1}^{n}\left(1-\frac{k}{n}\right)\rho_{k}.$$

**Figure 5.**
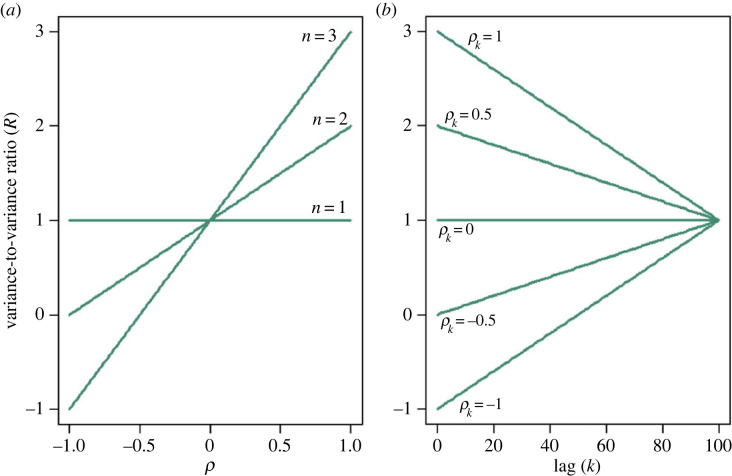
Variance-to-variance ratio *R*(*ρ*_1_, …, *ρ*_*k*_) with different values of the parameters *ρ*_*k*_, *n*, and *k*. (*a*) *R*(*ρ*, …, *ρ*) for *n* = 1, 2, 3. (*b*) *R*(0, …, 0, *ρ*_*k*_, 0, …, 0) for *k* = 1, …, *n* and *ρ*_*k*_ = −1, − 0.5, 0, 0.5, 1.

In general, positive autocorrelation is related to an increase in the variance of the mean and negative autocorrelation to a decrease in the variance of the mean. See figure 5 for some examples on how *R*(*ρ*_1_, …, *ρ*_*k*_) depends on its parameters.

**Example 4.1**

Suppose that *ρ*_*k*_ = *ρ* for *k* = 1, …, *n*. In this case for *ρ* = 0.05 and *n* = 101, we have *R* = 6, which means that, if we do not account for autocorrelation in the time series, we underestimate the variance of the sample mean by 6 times. Instead, if *ρ* = −0.01 and *n* = 11, then *R* = 0.9, which means that, without accounting for autocorrelation in the time series, the variance of the sample is overestimated by 10%. Note that the formula for *R* would not capture the full variation if *ρ*_*k*_ = *ρ* for every k∈N, as the time series would not be stationary [82].

**Example 4.2**

Suppose that *ρ*_*k*_ = *ρ* for one single *k* and zero otherwise. In this case, for *ρ* = 0.5, *n* = 80 and *k* = 20, we have *R* = 7/4, which means that, if we do not account for autocorrelation in the time series, the variance of the sample mean is underestimated by 75%. Instead, if *ρ* = −0.5 and *n* = 60 and *k* = 10, then *R* = 1/3, which means that, without accounting for autocorrelation in the time series, we overestimate the variance of the sample mean by 67%.

**Example 4.3**

If we consider subject 2 at *v* = 2.5 m s^−1^ from our dataset and the measurements of *k*_OLS_ (*n* = 75 strides), the only significant autocorrelation is at lag *k* = 14 with *ρ*_14_ = 0.2408617. If we use the formula for *R* with only one correlation lag different from zero, we get *R* = 1.391802. Therefore, neglecting *in toto* the autocorrelation structure of the time series would fail to account for nearly 40% of the variance of the average stiffness estimate across strides, even with one single lag showing significant correlation.

**Example 4.4**

If we consider subject 27 at *v* = 4.5 m s^−1^ from our dataset and the measurements of *k*_max_ (*n* = 89 strides), and consider only the largest significant lag in the first 40 lags, namely lag *k* = 8 with *ρ*_8_ = 0.2111892, we get *R* = 1.384412. Therefore, neglecting *in toto* the autocorrelation structure of the time series would again fail to account for nearly 40% of the variance of the average stiffness estimate across strides.

**Example 4.5**

If we consider subject 20 at *v* = 2.5 m s^−1^ from our dataset and the measurements of *k*_OLS_ (*n* = 88 strides), and consider all possible lags, we get *R* = 0.4162655. Therefore, neglecting *in toto* the autocorrelation structure of the time series would overestimate the variance of the average stiffness estimate across strides by nearly 60%.

**Example 4.6**

If we consider subject 26 at *v* = 3.5 m s^−1^ (*n* = 79 strides) from our dataset and the measurements of *k*_max_, we get the coefficient *R* = 0.3135405, which implies an overestimate of the variance of the average stiffness across strides of almost 70%.

We illustrated the risk of ignoring the correlation structure in the measurements using these several examples. The most direct consequence lies in mis-evaluating the magnitude of the variability of the estimate of the average stiffness across strides. Note that in the iid case, it is well known that the variance of the sample mean decreases on the order of 1/*n*, with *n* the sample size [67]. With correlation, things can go better or worse [82]. Positive correlation is associated with an increase in the variance (observations tend to depart from the mean and show stochastic trend), while negative correlation is associated with a decrease in the variance (observations tend to oscillate around the mean and stay at a bounded distance from the mean over time). In our examples, we show that ignoring correlation between strides can result in either scenario. Underestimating the variance can cause spurious results (e.g. not being able to assume independence of strides), whereas overestimating the variance can prevent the detection of the fixed effects that often motivate the study in the first place (e.g. stride and/or speed association with stiffness).

One of the consequences of broad impact of the mis-estimation of the variance is the difficulty in combining the results of different laboratories. If the variance of an average measurement of a variable of interest in one experiment at one single laboratory is miscalculated, the effect could be catastrophic when results are combined across laboratories [83,84]. This is the so-called *batch effect* and it is a major and interesting problem in machine learning. Often such a problem is addressed using transfer learning [85–87], a subfield of machine learning which aims at using information extracted from one population of interest for another population of interest. Further research in this direction is required in order to mitigate possible batch effects and study replicability problems in biomechanics. These problems have not received in biomechanics the attention that they have received in other fields, such as epidemiology, public health, neuroscience and genetics [83,88,89]. More research on uncertainty quantification (e.g. estimation of the variance), together with amassing large repositories of publicly available data, could potentially facilitate the interaction of multiple laboratories and lead to faster research progress in biomechanics and locomotion.

Altogether, this analysis underlines the necessity of determining the correlation structure across strides before modelling or performing hypothesis tests. Recall that if two random variables computed by different samples are dependent, then also the samples must be dependent [67]. Therefore, the dependence of stiffness on stride indicates a dependence of the stance phases themselves within experimental trials. This is quite important information as many biomechanical studies do not perform a dependency analysis before further testing and before assuming independence. It can happen, but it is quite a ‘rare’ occurrence, that two random variables computed from the same sample (and that are not constructed using only one distinct element of the sample each) are independent, even if the single observations of the sample are independent. This is clearly described with rigorous mathematical theorems in [67] and it depends on a classical result in statistical inference called Basu’s theorem [90]. In simple terms, the theorem states that under some regularity assumptions, minimal sufficient statistics for a parameter are independent of any ancillary statistics for that parameter. For example, the normal distribution is characterized by the condition that the sample mean (which is minimal sufficient for the population mean) is independent of the sample variance (which is ancillary for the population mean). With the word ‘characterized’ we mean that no other distribution possesses this property.

### 4.4. Overground experiments and the case of few strides per bout

In our analysis, we discussed to what extent autocorrelation between consecutive strides within a given trial of an experiment is present. We did not discuss extensively what happens to the variability of a measurement like leg stiffness in the case a researcher decides to estimate the parameter using a single stride per bout and to use multiple bouts. Using strides from different trials might require assessments such as: which stride of each bout shall we select? The one in the middle? Why? Is there a way to fairly compare bouts that have different numbers of strides? We believe that the answers to these questions require further data-driven research. Still, the strategy of using only one stride per bout risks to be sub-optimal because it does not seem reasonable to decide *a priori* to throw away a significant portion of the data merely because there might be a statistically yet-to-be-confirmed dependency across strides or because one does not want to deal with the dependency structure. Even just considering the experimental effort and the cost in terms of time, it is important to get the most out of the data collected. As shown in this manuscript, the statistical tools to harness stride dependency and to give a correct uncertainty quantification of the estimate of the parameter of interest are available. We recommend assessing the dependency structure of the sample also in the case in which an investigator decides to choose one single stride per bout and consider multiple bouts, but also in every other estimation method used. Again, failing to correctly assess the variability of a measurement (e.g. stiffness) might result in spurious findings or in losing the possibility to uncover important biological information. The determination of what the greatest sources of dependency (e.g. variation across strides versus variation across trials) are can have an important impact on the design of locomotion experiments and surely deserves further attention.

If multiple strides are available, it is really valuable information and, unless there has been some experimental failure owing to which some strides need to be excluded, it is important to keep them and evaluate them. We potentially understand the use of a single stride in the case in which the study is concentrated on that single stride, but even in such a case there is something to say. Suppose that a researcher wants to understand the behaviour of individuals during the first stride of a locomotor trial. Then, there is one single first stride in each experiment. Especially if the strides are independent, it is a reasonable choice to use only the first stride of the experiment for any estimate related to the first stride. However, the situation here is also tricky because if there is autocorrelation at lag 1, some information about the first stride is shadowed in the second stride, too. In a similar way, there is potentially some information about the first stride in other strides as well. Note that autocorrelation at lag 1 does not mean that stride 1 is correlated to stride 2 or that stride 2 is correlated to stride 3 on an individual stride-by-stride basis. Rather, it means that there is correlation between consecutive strides globally in the data. In the simplest autoregressive model AR(1) [82], there are two parameters: the variance of the noise and the correlation coefficient *ρ* between one observation and the next. In such a case, *ρ*_*k*_ = *ρ*^*k*^, with *k* the lag considered. This means that the correlation predicted by the AR(1) model decays exponentially with the lag, but it is present at every lag. If, for example, the correlation is 0.7 at lag 1, at lag 2 the correlation is 0.49. The implication of this is that, removing only a bunch of strides from the sample might not remove in full the autocorrelation among the remaining strides. The case of correlation at large lags is a statistically more interesting case, but also very interesting from the biomechanical perspective because, although one might intuitively expect a relationship from one stride to the next, one might not expect to see a relationship between one stride and strides that take place 5 to 10 strides later.

It is more common for data containing large numbers of strides to be available during running on a treadmill than overground or on uneven ground [48]. Therefore, the implications of the present study may be most relevant to treadmill-based locomotion studies. In overground studies with stationary trackways and force plates, the animal moves across the field of view just once and so the number of strides per bout available for analysis is more limited. The dataset that we analysed [28] is of treadmill running and so we concentrated more on uncertainty quantification in that type of experiment. However, parts of our discussion also apply to the case in which fewer strides are available. In fact, although we highlight in figure 1 those subjects whose strides showed autocorrelation at large lags (we put a threshold at lag 5), figure 1 shows that autocorrelation was also present at lower lags.

If relatively few strides are available (e.g. overground running), then the variance can be computed only using those strides, with calculations analogous to those in the examples of §4.3 (e.g. example 2, but with a smaller lag). Still, if there is dependence between one stride and another, many strides can provide information about many others. As noted in our analysis, some strides might be outliers. If it is determined that a stride is an outlier via a careful verification with a test for outliers, the exclusion of that stride is recommended. Care must be taken, especially in the case in which only a few strides are available. A popular test for outliers is Grubbs’s test [91], but such a test gives reasonable results only when there are enough observations, as makes intuitive sense. In the case in which too few strides are available, Grubbs’s test will, most likely, overcount outliers. It is a general fact that when the sample is small, the estimation of the variance has limited validity. The extreme case of considering one stride per trial is essentially kind of equivalent to implicitly assuming that there is no variability across strides. In this paper, we provided evidence that such an assumption might be invalid. When it is not necessary to consider only one single stride per trial, such a decision should not be made lightly because there is no physical principle or mathematical law that explains the relationship between the value of many locomotor parameters like stiffness and the stride number in a locomotor bout. The problem of correctly extracting information about the variability of an estimate in overground locomotion may be first experimental, and relate to data collection, before potentially being statistical. In the case of human locomotion, until the instrumentation to record more than a few dozen metres of overground running is widely available, then researchers will always have relatively few strides available for study and comparatively little information about uncertainty quantification for parameters of interest. In turn, this will negatively impact the replicability of overground studies across laboratories.

### 4.5. Other considerations

For what concerns the estimation of stiffness *in se*, there is always the question: what stiffness are we really estimating? [42,43]. As far as we know, there is no mathematical model that describes in a satisfactorily quantitative way the process through which the brain modulates leg stiffness. Reductionist models, such as SLIP, describe the most essential locomotor dynamics with the most parsimonious model in terms of the number of variables used [29,30,80]. In our *model 1*—*k*_max_, we used a method which is biologically and mechanically well-motivated by the very definition of spring constant and elastic force. In our *model 2*—*k*_OLS_, we considered the stiffness as the least square estimates of a simple linear regression model without intercept [92]. Note that this second method might be criticizable for the possibly more cumbersome biological interpretation. However, the first method, as well, is questionable from the biological perspective, as the stiffness is not associated with the stiffness of any specific tissue, but it is a resulting parameter of a complex system which collapsed from a multi-legged body with distributed mass to a single point mass on a spring [80]. We believe that *k*_max_ and *k*_OLS_ are good examples for the purposes of our paper, as they emerge from quite alternative perspectives (biologically driven *k*_max_ and data-driven *k*_OLS_). Incorporating a broad comparison of all possible methods for calculating stiffness is beyond the scope of this paper. Such an analysis would possibly encounter a similar across-strides-dependence problem and possibly lead to analogous results and consequences.

**Figure 7.**
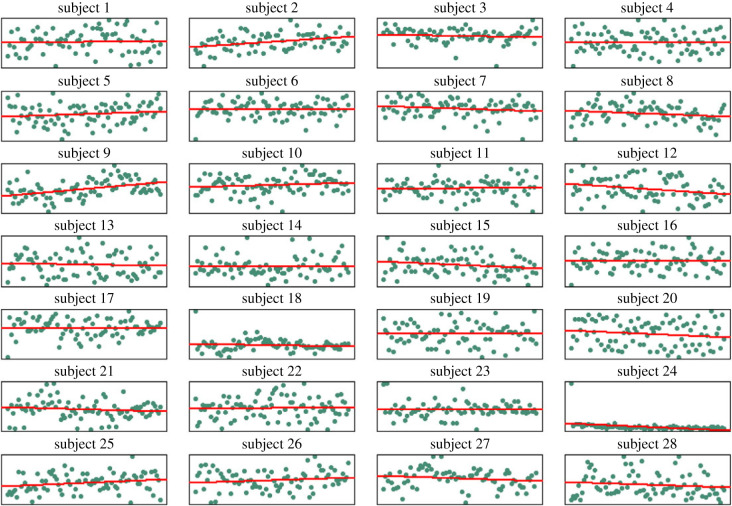
Deterministic trend of *k*_OLS_ across strides for each of the 28 subjects at *v* = 2.5 m s^−1^.

**Figure 8.**
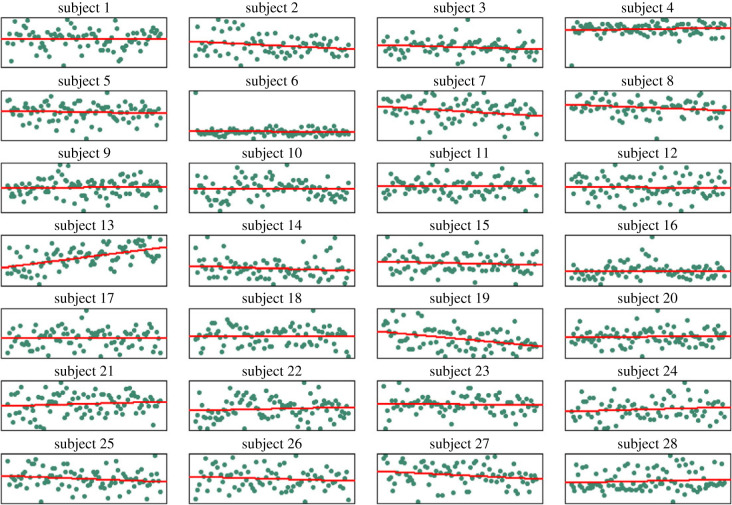
Deterministic trend of *k*_OLS_ across strides for each of the 28 subjects at *v* = 3.5 m s^−1^.

**Figure 9.**
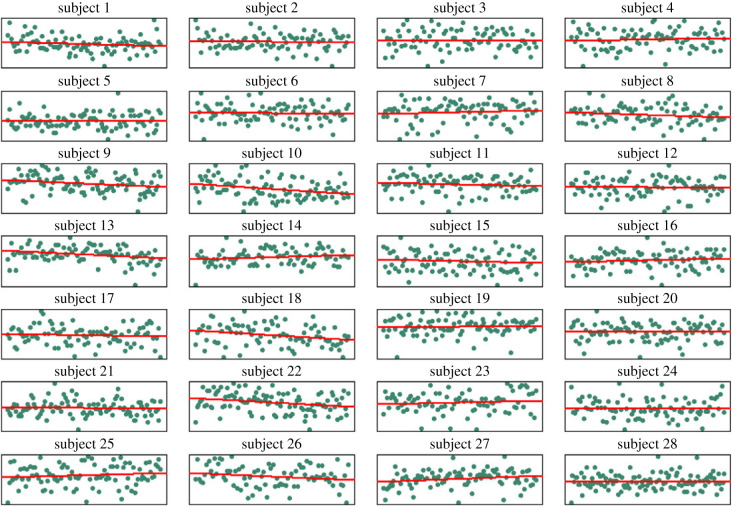
Deterministic trend of *k*_OLS_ across strides for each of the 28 subjects at *v* = 4.5 m s^−1^.

**Figure 10.**
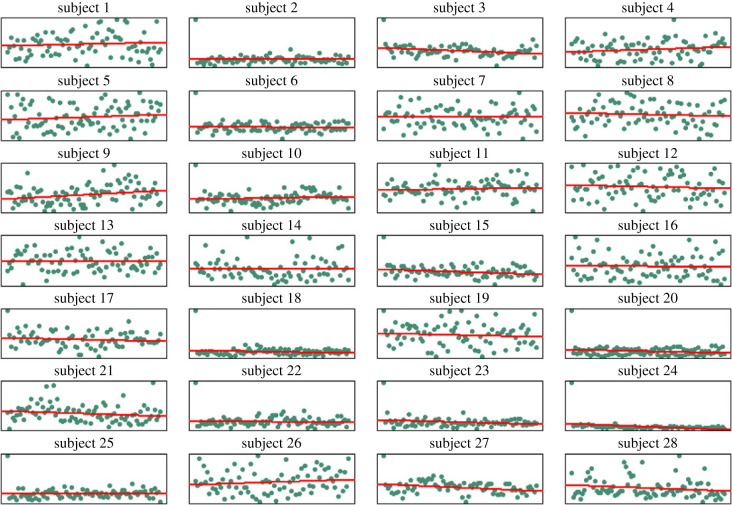
Deterministic trend of *k*_max_ across strides for each of the 28 subjects at *v* = 2.5 m s^−1^.

**Figure 11.**
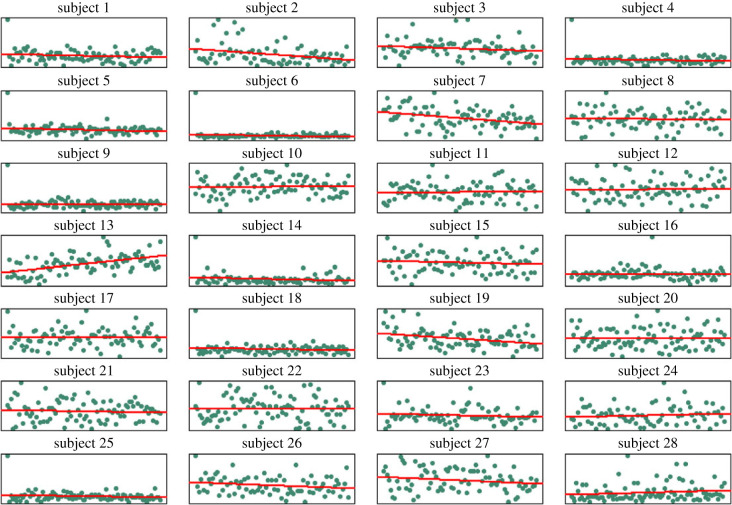
Deterministic trend of *k*_max_ across strides for each of the 28 subjects at *v* = 3.5 m s^−1^.

**Figure 12.**
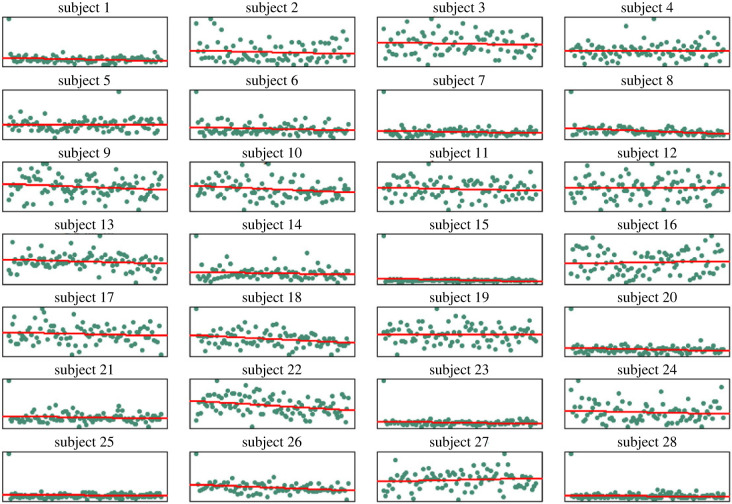
Deterministic trend of *k*_max_ across strides for each of the 28 subjects at *v* = 4.5 m s^−1^.

Note that for several of the subjects, there is a possible outlier in the stiffness measurements of the first strides (figures 7–12). We did not remove those observations before fitting the model, as it is actually relevant to know that, in some subjects, the first stride needs to be tested as a possible outlier. As mentioned in the introduction, biomechanists often address this first-stride-problem by removing the first and last strides of an experiment from the analysed dataset [1]. Sometimes those strides are not even recorded because they occur off camera. Again, we consider removal strategies suboptimal; in this case, removing initial and final strides compromises the opportunity to model the initial transition phase. Depending on the study, researchers might not be interested in the opportunity of modelling transitional phases. In such cases, first and last strides are removed from consideration because they might contain a noisy signal. Note that, also in such cases, careful statistical considerations must be made before deciding on the removal of those strides. How many strides should be removed? This is a tricky question. In some cases, after the removal of an outlier with the support of a rigorous statistical test, the same test (e.g. Grubbs’s test [91]) on the reduced dataset (without the observation that was determined to be an outlier after the first test) might detect an additional outlier that was not detected when the test was initially performed on the full dataset. Therefore, we recommend to thoughtfully test for outliers also in studies of steady-state locomotion, before making the decision to remove some strides from the data, and to quantitatively support the decision to consider some strides transitional. If the first or last strides are not outliers, there is not really a statistical justification in support of removing such observations from the analysis. Although the removal of first and last strides is perceived as safe and convincing from a biomechanical perspective, if those strides are not outliers, curtailing the data in this way will end up having a negative impact on the correct estimation of variability, with the worst effects occurring when the number of available strides is lowest (e.g. experiments taking place overground or on uneven ground).

## 5. Conclusion

As far as we know, this is the first paper in the terrestrial biomechanics literature dedicated to discussing rigorously and extensively the effects of neglecting the correlation structure of consecutive strides. We found evidence of autocorrelation of the time series of human leg stiffness across strides during running on a treadmill and, thereby, evidence of the dependence of the time series of kinematic and kinetic data at different strides. Therefore, our paper has implications for the diffuse practice in biomechanics of assuming that individual strides within an experimental trial are independent and identically distributed. Lacking an understanding of stride dependence structure invalidates estimates of uncertainty of average measurements across strides, including the variance. Variability assessments are required for both building correct confidence intervals and valid hypothesis testing. The incorrect quantification of uncertainty might inflate biological signals in some cases or miss it in others. We caution against the practice of blindly assuming stride independence and recommend that researchers first test for stride independence before assuming it. If, after testing, the strides turn out to not be independent, we recommend exploiting the correlation structure. By contrast, retaining only one of every few strides is a suboptimal remedy because it decreases the sample size by an order of magnitude and risks to disrupt the correlation structure of the time series collected from the experiment. Having an incorrect estimate of the variability of biologically important quantities such as leg stiffness or any other measurement can negatively impact the replicability of the study. We argue that it is important not only to include measurements of uncertainty peculiar to an experiment in every study, but also to have a standardized methodology to assess uncertainty. Not only would this facilitate replicability and comparison, but it would also foster collaboration across research groups.

## Figures and Tables

**Figure 6.**
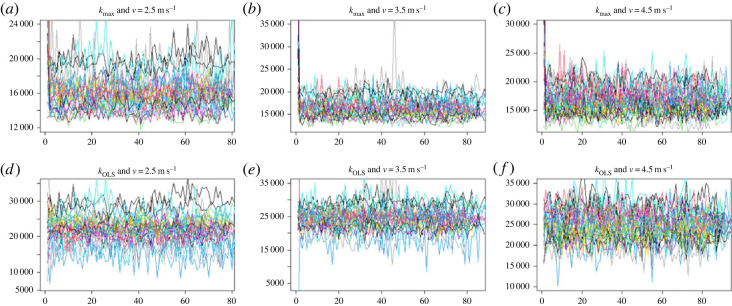
Trajectories of *k*_max_ (*a*–*c*) and *k*_OLS_ (*d*–*f*) of each subject across the three speeds considered *v* = 2.5 m s^−1^ (*a*,*d*), 3.5 m s^−1^ (*b*,*e*) and 4.5 m s^−1^ (*c*,*f*) for all the recorded strides. The *y*-axes represent stiffness *k* and the *x*-axes represent the stride number.

**Figure 13.**
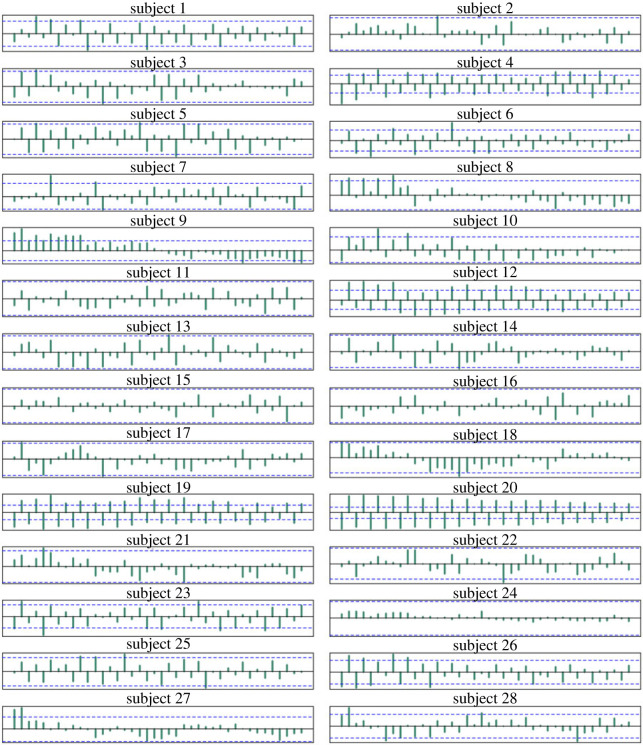
Autocorrelation function of *k*_OLS_ across strides for each of the 28 subjects at *v* = 2.5 m s^−1^. When a green vertical bar exceeding the blue dotted line in either positive or negative directions, it indicates the presence of significant autocorrelation at that lag.

**Figure 14.**
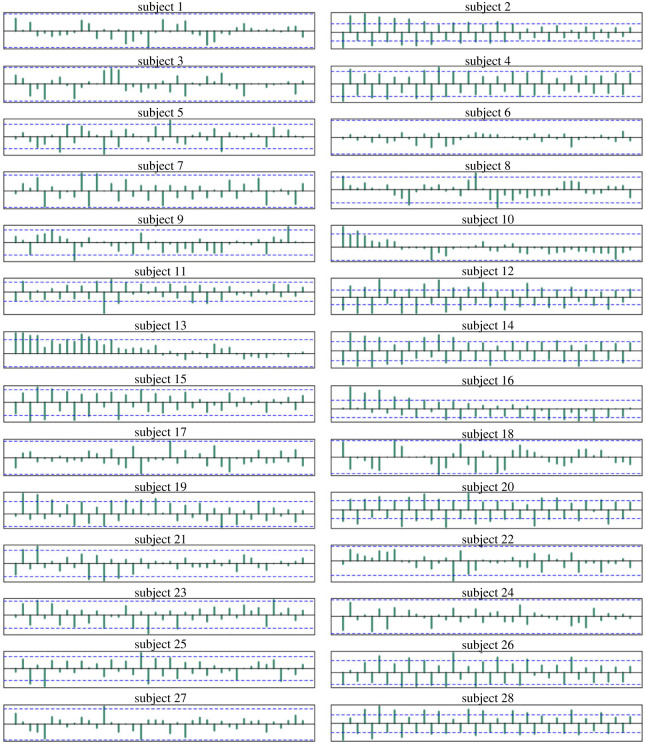
Autocorrelation function of *k*_OLS_ across strides for each of the 28 subjects at *v* = 3.5 m s^−1^. When a green vertical bar exceeding the blue dotted line in either positive or negative directions, it indicates the presence of significant autocorrelation at that lag.

**Figure 15.**
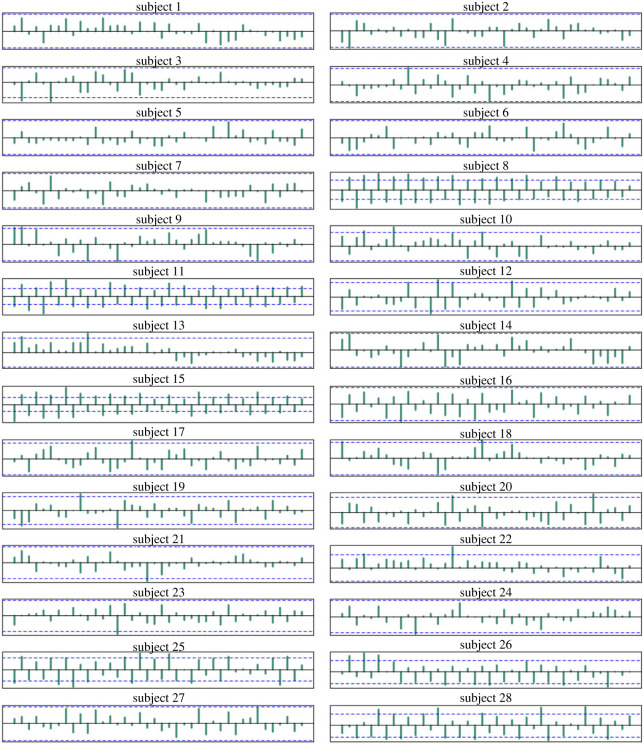
Autocorrelation function of *k*_OLS_ across strides for each of the 28 subjects at *v* = 4.5 m s^−1^. When a green vertical bar exceeding the blue dotted line in either positive or negative directions, it indicates the presence of significant autocorrelation at that lag.

**Figure 16.**
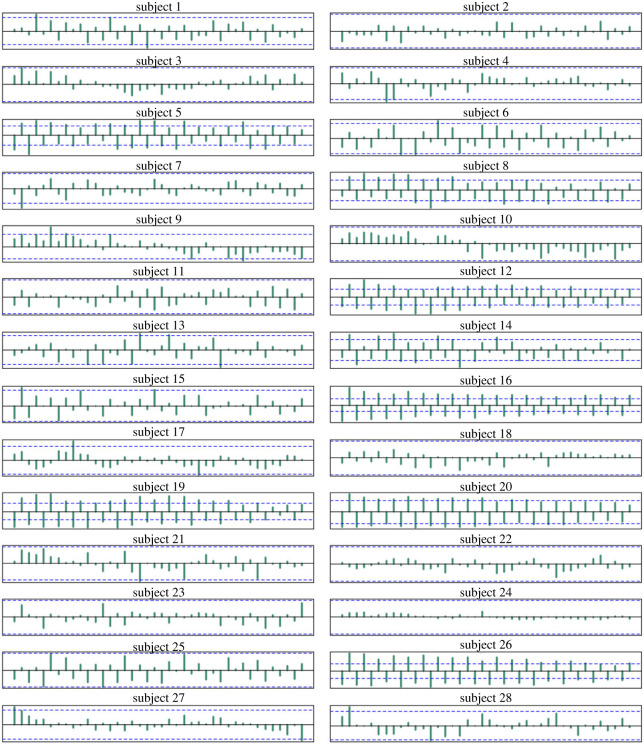
Autocorrelation function of *k*_max_ across strides for each of the 28 subjects at *v* = 2.5 m s^−1^. When a green vertical bar exceeding the blue dotted line in either positive or negative directions, it indicates the presence of significant autocorrelation at that lag.

**Figure 17.**
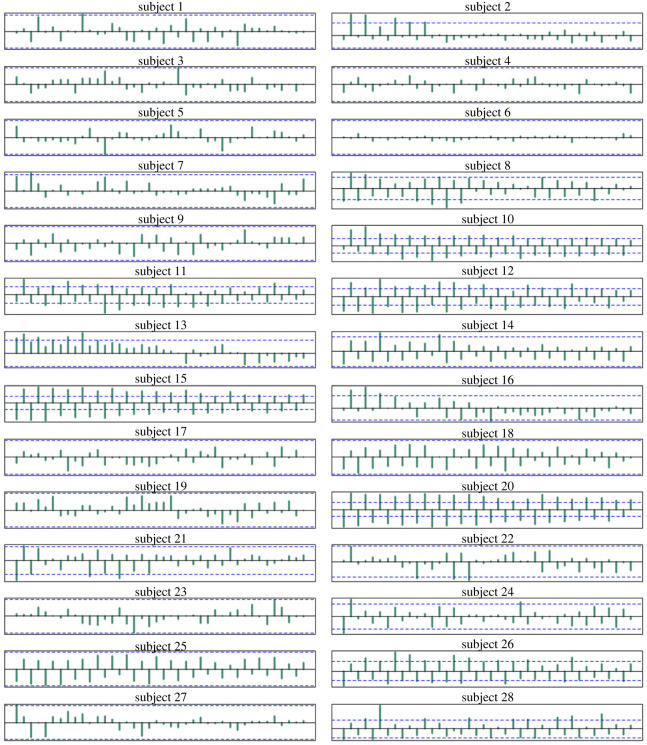
Autocorrelation function of *k*_max_ across strides for each of the 28 subjects at *v* = 3.5 m s^−1^. When a green vertical bar exceeding the blue dotted line in either positive or negative directions, it indicates the presence of significant autocorrelation at that lag.

**Figure 18.**
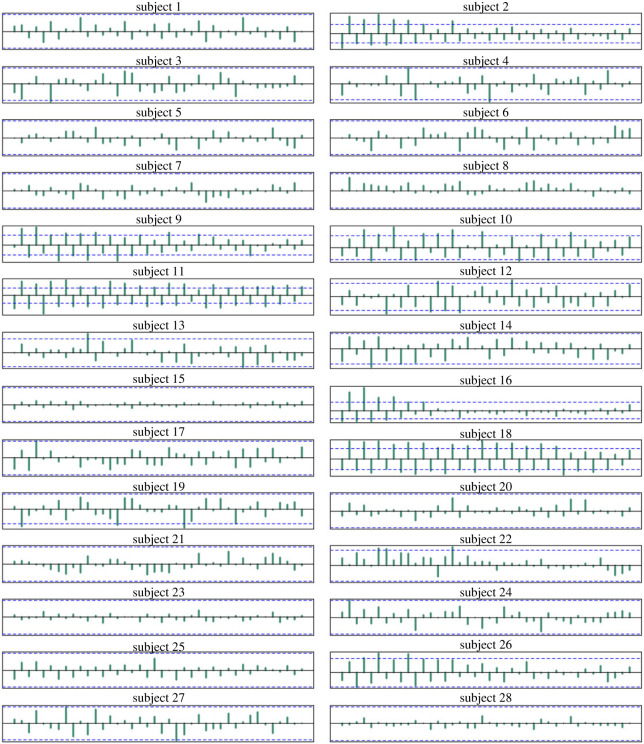
Autocorrelation function of *k*_max_ across strides for each of the 28 subjects at *v* = 4.5 m s^−1^.

## Data Availability

Data can be accessed at the website; https://figshare.com/articles/dataset/A_comprehensive_public_data_set_of_running_biomechanics_and_the_effects_of_running_speed_on_lower_extremity_kinematics_and_kinetics/4543435; and code via the electronic supplementary material [93].

## Appendix A

This appendix contains results of some additional analyses that we performed.

**A.1. Stiffness trajectories at different speeds**

Figure 6 below shows the different trajectories of *k*_OLS_ and *k*_max_ for all the recorded stride of every subject for the three speeds considered *v* = 2.5 m s^−1^, 3.5 m s^−1^and 4.5 m s^−1^.

**A.2. Deterministic trend of stiffness across strides per subject**

In this section, we checked for the presence of a deterministic trend of human leg stiffness across strides using both the methods for calculating stiffness (*k*_OLS_ and *k*_max_). The models we fitted were

$$k_{i}=\beta_{0}+\beta_{1}S_{i}+\epsilon_{i},\;i=1,\ldots ,28,$$

with *k*_*i*_ being *k*_OLS_ and *k*_max_ and *S*_*i*_, *i* = 1, …, 28 being the stride number. In total, we had six of these models, one for each combination of velocity (*v* = 2.5 m s^−1^, *v* = 3.5 m s^−1^ and *v* = 4.5 m s^−1^) and stiffness *k* (*k* = *k*_OLS_ and *k* = *k*_max_). The level of significance was *α* = 0.05 and *p*-values were calculated with 4 decimals of significance. In some of the subjects and for some of the conditions and measurements of stiffness, there was what appeared to be an outlier at the beginning of the stride considered. As mentioned in the core part of this manuscript (§4.5), we decided to keep that first observation as it was significant for our discussion, given that its presence further supports the need to consider dependence and variability across strides.

The analysis of the deterministic trend, always modelled as linear in our models, showed some significant results across conditions and subjects for both stiffness measurements *k*_OLS_, *k*_max_. The significance of this result is not that we determined that the stiffness changes across stride linearly, but that there might be such a change, and it is risky to neglect such a relationship.

*k*_OLS_ and *v* = 2.5 m s^−1^

There is a significant correlation between stride *S* and stiffness *k*_OLS_ in 5 out of 28 subjects in the case *v* = 2.5m s^−1^ (figure 7 and table 3).

**Table 3.** The *p*-values produced when testing for the presence of a significant deterministic linear relationship between the stride number and *k*_OLS_ for each of the 28 subjects at *v* = 2.5 m s^−1^. (Bold indicates *p* < 0.05.)

subject 1	subject 2	subject 3	subject 4
0.8411	**0.0041**	0.3339	0.7724
subject 5	subject 6	subject 7	subject 8
0.1727	0.6668	0.1108	0.1182
subject 9	subject 10	subject 11	subject 12
**0.0001**	0.1243	0.7434	**0.0163**
subject 13	subject 14	subject 15	subject 16
0.5029	0.7842	0.0688	0.9185
subject 17	subject 18	subject 19	subject 20
0.9087	0.4223	0.9920	0.2044
subject 21	subject 22	subject 23	subject 24
0.2409	0.6083	0.7839	**0.0025**
subject 25	subject 26	subject 27	subject 28
**0.0382**	0.2333	0.1686	0.2378

*k*_OLS_ and *v* = 3.5 m s^−1^

There is a significant correlation between stride *S* and stiffness *k*_OLS_ in 4 out of 28 subjects in the case *v* = 3.5 m s^−1^ (figure 8 and table 4).

**Table 4.** The *p*-values produced when testing for the presence of a significant deterministic linear relationship between the stride number and *k*_OLS_ for each of the 28 subjects at *v* = 3.5 m s^−1^. (Bold indicates *p* < 0.05.)

subject 1	subject 2	subject 3	subject 4
0.8348	0.0560	0.2325	0.3368
subject 5	subject 6	subject 7	subject 8
0.4128	0.2853	bf 0.0168	0.0581
subject 9	subject 10	subject 11	subject 12
0.6165	0.9244	0.9203	0.7140
subject 13	subject 14	subject 15	subject 16
**<0.0001**	0.2614	0.3294	0.6886
subject 17	subject 18	subject 19	subject 20
0.7568	0.7385	**0.0002**	0.6169
subject 21	subject 22	subject 23	subject 24
0.2495	0.4178	0.7642	0.3234
subject 25	subject 26	subject 27	subject 28
0.1185	0.2968	**0.0483**	0.3173

*k*_OLS_ and *v* = 4.5 m s^−1^

There is a significant correlation between stride *S* and stiffness *k*_OLS_ in 5 out of 28 subjects in the case *v* = 4.5 m s^−1^ (figure 9 and table 5).

**Table 5.** The *p*-values produced when testing for the presence of a significant deterministic linear relationship between the stride number and *k*_OLS_ for each of the 28 subjects at *v* = 4.5 m s^−1^. (Bold indicates *p* < 0.05.)

subject 1	subject 2	subject 3	subject 4
0.8411	**0.0041**	0.3339	0.7724
subject 5	subject 6	subject 7	subject 8
0.1727	0.6668	0.1108	0.1182
subject 9	subject 10	subject 11	subject 12
**0.0001**	0.1243	0.7434	**0.0163**
subject 13	subject 14	subject 15	subject 16
0.5029	0.7842	0.0688	0.9185
subject 17	subject 18	subject 19	subject 20
0.9087	0.4223	0.9920	0.2044
subject 21	subject 22	subject 23	subject 24
0.2409	0.6083	0.7839	**0.0025**
subject 25	subject 26	subject 27	subject 28
**0.0382**	0.2333	0.1686	0.2378

*k*_max_ and *v* = 2.5 m s^−1^

There is a significant correlation between stride *S* and stiffness *k*_max_ in 5 out of 28 subjects in the case *v* = 2.5 m s^−1^ (figure 10 and table 6).

**Table 6.** The *p*-values produced when testing for the presence of a significant deterministic linear relationship between the stride number and *k*_max_ for each of the 28 subjects at *v* = 2.5 m s^−1^. (Bold indicates *p* < 0.05.)

subject 1	subject 2	subject 3	subject 4
0.4520	0.6103	**0.0162**	0.1165
subject 5	subject 6	subject 7	subject 8
0.2355	0.4618	0.9171	0.4191
subject 9	subject 10	subject 11	subject 12
**0.0184**	0.3564	0.4658	0.4379
subject 13	subject 14	subject 15	ssubject 16
0.6891	0.9220	**0.0176**	0.6669
subject 17	subject 18	subject 19	subject 20
0.2488	0.2617	0.4096	0.1655
subject 21	subject 22	subject 23	subject 24
0.1410	0.4899	0.0862	**0.0083**
subject 25	subject 26	subject 27	subject 28
0.8918	0.2867	**0.0119**	0.1215

*k*_max_ and *v* = 3.5 m s^−1^

There is a significant correlation between stride *S* and stiffness *k*_max_ in 4 out of 28 subjects in the case *v* = 3.5 m s^−1^ (figure 11 and table 7).

**Table 7.** The *p*-values produced when testing for the presence of a significant deterministic linear relationship between the stride number *S* and *k*_max_ for each of the 28 subjects at *v* = 3.5 m s^−1^. (Bold indicates *p* < 0.05.)

subject 1	subject 2	subject 3	subject 4
0.2106	**0.0060**	0.1172	0.1994
subject 5	subject 6	subject 7	subject 8
0.0829	0.2021	**0.0012**	0.5693
subject 9	subject 10	subject 11	subject 12
0.4309	0.6504	0.7665	0.6102
subject 13	subject 14	subject 15	subject 16
**<0.0001**	0.1913	0.3728	0.6726
subject 17	subject 18	subject 19	subject 20
0.8171	0.2153	**0.0031**	0.7889
subject 21	subject 22	subject 23	subject 24
0.5151	0.7966	0.2783	0.2989
subject 25	subject 26	subject 27	subject 28
0.1207	0.0742	0.1075	0.1732

*k*_max_ and *v* = 4.5 m s^−1^

There is a significant correlation between stride *S* and stiffness *k*_max_ in 5 out of 28 subjects in the case *v* = 4.5 m s^−1^ (figure 12 and table 8).

**Table 8.** The *p*-values produced when testing for the presence of a significant deterministic linear relationship between the stride number *S* and *k*_max_ for each of the 28 subjects at *v* = 4.5 m s^−1^. (Bold indicates *p* < 0.05.)

subject 1	subject 2	subject 3	subject 4
0.0601	0.4452	0.4808	0.5843
subject 5	subject 6	subject 7	subject 8
0.7060	0.0906	0.1709	**0.0005**
subject 9	subject 10	subject 11	subject 12
0.1151	**0.0253**	0.2640	0.9275
subject 13	subject 14	subject 15	subject 16
0.0989	0.3569	0.0777	0.5435
subject 17	subject 18	subject 19	subject 20
0.2608	**0.0045**	0.7787	0.1241
subject 21	subject 22	subject 23	subject 24
0.2418	**0.0042**	0.1774	0.3052
subject 25	subject 26	subject 27	subject 28
0.2701	**0.0042**	0.2739	0.1942

**A.3. Autocorrelation of stiffness across strides per subject**

In this subsection, we computed and plotted the sample ACF ${\hat{\rho }}_{Y}(k)\;:={\hat{\gamma }}_{Y}(k)/{\hat{\gamma }}_{Y}(0)$ to estimate the ACF *ρ*_*Y*_(*k*) : = Corr(*Y*_*t*_, *Y*_*t*+*k*_) for *k* = 1, … , 40 and both measures of stiffness ($k_{\mathrm{OLS}}\text{ and }k_{\max}$) for each subject *i* = 1, … , 28 and velocity *v* = 2.5 m s^−1^, *v* = 3.5 m s^−1^ and *v* = 4.5 m s^−1^. Our main goal was to detect if the observations $k_{\mathrm{OLS}}\text{ and }k_{\max}$, measured at different strides, are iid or not. When *k* starts to be only slightly smaller than *n*, the estimates ${\hat{\rho }}_{Y}(k),{\hat{\gamma }}_{Y}(k)$ are unreliable since there are only a few observations to use for the estimates. Therefore, we stopped the lag at 40, which was reasonably less than the minimum (across subjects) number of strides observed.

Note that *ρ*_*Y*_(*k*) gives the full description of the covariance structure of the time series *Y*_*t*_ only in the stationary case, but not in the non-stationary case. In the non-stationary case, the ACF depends on both the lag and the position of the random variables in the time-series order [82]. We do not enter here in the full analysis of what more can go wrong in the case in which the time series of strides are non-stationary (to keep the paper of a reasonable length, we did not include this analysis for our data). In the non-stationary case, the iid hypothesis can be violated also in the case in which observations are independent (and so they must be not identically distributed). This happens, for example, for a time series $Y_{t}∼N(0,\exp(t))$ with $Y_{t_{1}}$ independent of $Y_{t_{2}}$ for every *t*_1_ ≠ *t*_2_. Note that, in this case, the variance is growing exponentially in time, and so, naturally, later observations are more dispersed than earlier observations. This needs to be taken into account for an accurate quantification of uncertainty. Recall, that a way to understand if a time series is not stationary is by checking if *ρ*_*Y*_(*k*) → 0. Indeed, it is well known (See Proposition 2.2 [82]) that for causal ARMA processes, *ρ*_*Y*_(*k*) → 0 at an exponential rate as |*k*| → +∞. By Wold decomposition theorem, a purely non-deterministic Gaussian process is an *MA*(∞) process with normal white noise (See Proposition 2.2 [82]). This argument tells us that a lack of fast decay in the ACF, is a hint of non-stationarity of the process. The lack of decay to zero of the ACF implies an even further distance from zero of the ACF. Therefore, non-stationarity implies an even stronger deviation from the iid hypothesis [82] and so a further complication for the analysis of uncertainty. As it is depicted in figures 13–18, the analysis of the autocorrelation function through strides for $k_{\mathrm{OLS}}\text{ and }k_{\max}$ for each subject at all velocity conditions *v* = 2.5 m s^−1^, *v* = 3.5 m s^−1^ and *v* = 4.5 m s^−1^ indicates that, potentially, some of the time series were non-stationary [82].

*k*_OLS_ and *v* = 2.5 m s^−1^ The ACF of 9 out of 28 subjects does not seem to decay, indicating that the time series might be non-stationary.

*k*_OLS_ and *v* = 3.5 m s^−1^ The ACF of 9 out of 28 subjects does not seem to decay, indicating that the time series might be non-stationary.

*k*_OLS_ and *v* = 4.5 m s^−1^ The ACF of 5 out of 28 subjects does not seem to decay, indicating that the time series might be non-stationary.

*k*_max_ and *v* = 2.5 m s^−1^ The ACF of 7 out of 28 subjects does not seem to decay, indicating that the time series might be non-stationary.

*k*_max_ and *v* = 3.5 m s^−1^ The ACF of 9 out of 28 subjects does not seem to decay, indicating that the time series might be non-stationary.

*k*_max_ and *v* = 4.5 m s^−1^ The ACF of 4 out of 28 subjects does not seem to decay, indicating that the time series might be non-stationary.

**A.4. Linear mixed effect models for each velocity, separately**

The tables below show the outputs of the LMEMs built for each velocity, separately. As for the LMEMs in which both velocity and stride were included as covariates, we fitted the models using the R-package *lmer* described in [79].

All models with *k*_max_ (tables 9–11) show statistically significant effect of stride, while only the condition *v* = 4.5 m s^−1^ for *k*_OLS_ (tables 12–14) shows a statistically significant dependence on stride (see also §3.3).

**Table 9.** Stride has a significant fixed effect on *k*_max_ at *v* = 2.5 m s^−1^. (Bold indicates *p* < 0.05.)

*k*_max_ and *v* = 2.5 m s^−1^: linear mixed effects model					
fixed effects	estimate	s.e.	d.f.	*t*-value	*p*-value
(intercept)	16218.903	330.554	28.402	49.066	**<0.0001**
stride	−4.125	1.281	2226.345	−3.221	**0.0013**

**Table 10.** Stride has a significant fixed effect on *k*_max_ at *v* = 3.5 m s^−1^. (Bold indicates *p* < 0.05.)

*k*_max_ and *v* = 3.5 m s^−1^: linear mixed effects model					
fixed effects	estimate	s.e.	d.f.	*t*-value	*p*-value
(intercept)	16635.470	338.322	28.750	49.171	**<0.0001**
stride	−4.589	1.380	2356.552	−3.325	**0.0009**

**Table 11.** Stride has a significant fixed effect on *k*_max_ at *v* = 4.5 m s^−1^. (Bold indicates *p* < 0.05.)

*k*_max_ and *v* = 4.5 m s^−1^: linear mixed effects model					
fixed effects	estimate	s.e.	d.f.	*t*-value	*p*-value
(intercept)	16218.903	330.554	28.402	49.066	**<0.0001**
stride	−4.125	1.281	2226.345	−3.221	**0.0013**

**Table 12.** Stride does not have a significant fixed effect on *k*_OLS_ at *v* = 2.5 m s^−1^. (Bold indicates *p* < 0.05.)

*k*_OLS_ and *v* = 2.5 m s^−1^: linear mixed effects model					
fixed effects	estimate	s.e.	d.f.	*t*-value	*p*-value
(intercept)	22439.317	651.818	28.024	34.426	**<0.0001**
stride	−1.633	2.164	2226.256	−0.754	0.451

**Table 13.** Stride does not have a significant fixed effect on *k*_OLS_ at *v* = 3.5 m s^−1^. (Bold indicates *p* < 0.05.)

*k*_OLS_ and *v* = 3.5 m s^−1^: linear mixed effects model					
fixed effects	estimate	s.e.	d.f.	*t*-value	*p*-value
(Intercept)	24304.192	545.335	28.384	44.567	**<0.0001**
Stride	−1.429	1.988	2356.439	−0.719	0.472

**Table 14.** Stride has a significant fixed effect on *k*_OLS_ at *v* = 4.5 m s^−1^. (Bold indicates *p* < 0.05.)

*k*_OLS_ and *v* = 4.5 m s^−1^: linear mixed effects model					
fixed effects	estimate	s.e.	d.f.	*t*-value	*p*-value
(intercept)	25089.457	588.680	28.009	42.620	**<0.0001**
stride	−3.984	1.724	2532.462	−2.311	**0.0209**
